# Oxidative and glycolytic skeletal muscles deploy protective mechanisms to avoid atrophy under pathophysiological iron overload

**DOI:** 10.1002/jcsm.12897

**Published:** 2022-02-03

**Authors:** David Martin, Kévin Nay, François Robin, Amélie Rebillard, Luz Orfila, Brice Martin, Patricia Leroyer, Pascal Guggenbuhl, Suzanne Dufresne, Philippe Noirez, Martine Ropert, Olivier Loréal, Frédéric Derbré

**Affiliations:** ^1^ Laboratory ‘Movement, Sport and Health Sciences’—EA7470 University of Rennes/ENS Rennes Bruz France; ^2^ Exercise and Nutrition Research Program, Mary MacKillop Institute for Health Research Australian Catholic University Melbourne Vic. Australia; ^3^ INSERM, INRAe, University of Rennes, Nutrition Metabolisms and Cancer Institute (NuMeCan), Platform AEM2, CHU Rennes Rennes France; ^4^ IRMES—Institute for Research in Medicine and Epidemiology of Sport, INSEP Paris France; ^5^ INSERM S1124, Université de Paris Paris France; ^6^ EA7507, Performance Health Metrology Society, Université de Reims Champagne Ardenne Reims France; ^7^ Département de Biochimie CHU Rennes Rennes France

**Keywords:** Sarcopenia, Disuse, Typology, Myosin heavy chain, Mitochondria

## Abstract

**Background:**

Iron excess has been proposed as an essential factor in skeletal muscle wasting. Studies have reported correlations between muscle iron accumulation and atrophy, either through ageing or by using experimental models of secondary iron overload. However, iron treatments performed in most of these studies induced an extra‐pathophysiological iron overload, more representative of intoxication or poisoning. The main objective of this study was to determine the impact of iron excess closer to pathophysiological conditions on structural and metabolic adaptations (i) in differentiated myotubes and (ii) in skeletal muscle exhibiting oxidative (i.e. the soleus) or glycolytic (i.e. the gastrocnemius) metabolic phenotypes.

**Methods:**

The impact of iron excess was assessed in both *in vitro* and *in vivo* models. Murine differentiated myotubes were exposed to ferric ammonium citrate (FAC) (i.e. 10 and 50 μM) for the *in vitro* component. The *in vivo* model was achieved by a single iron dextran subcutaneous injection (1 g/kg) in mice. Four months after the injection, soleus and gastrocnemius muscles were harvested for analysis.

**Results:**

*In vitro*, iron exposure caused dose‐dependent increases of iron storage protein ferritin (*P* < 0.01) and dose‐dependent decreases of mRNA TfR1 levels (*P* < 0.001), which support cellular adaptations to iron excess. Extra‐physiological iron treatment (50 μM FAC) promoted myotube atrophy (*P* = 0.018), whereas myotube size remained unchanged under pathophysiological treatment (10 μM FAC). FAC treatments, whatever the doses tested, did not affect the expression of proteolytic markers (i.e. NF‐κB, MurF1, and ubiquitinated proteins). *In vivo*, basal iron content and mRNA TfR1 levels were significantly higher in the soleus compared with the gastrocnemius (+130% and +127%; *P* < 0.001, respectively), supporting higher iron needs in oxidative skeletal muscle. Iron supplementation induced muscle iron accumulation in the soleus and gastrocnemius muscles (+79%, *P* < 0.001 and +34%, *P* = 0.002, respectively), but ferritin protein expression only increased in the gastrocnemius (+36%, *P* = 0.06). Despite iron accumulation, muscle weight, fibre diameter, and myosin heavy chain distribution remained unchanged in either skeletal muscle.

**Conclusions:**

Together, these data support that under pathophysiological conditions, skeletal muscle can protect itself from the related deleterious effects of excess iron.

## Introduction

Extreme physical inactivity, ageing, malnutrition, and many diseases (e.g. cancer, myopathy, and acute and chronic inflammatory diseases) are recognized to promote loss of skeletal muscle mass, decreases in strength, and increased fatigue.^1^ Muscle wasting is a major clinical issue that contributes to reduced autonomy and quality of life, leading to increased morbidity and mortality.^1^ As such, understanding the cellular mechanisms involved in muscle atrophy is critical to developing efficient prevention strategies for patients, medical team, and the healthcare system.^2^, ^3^ Muscle atrophy is characterized by the loss of myofibrils due to an imbalance between protein synthesis and proteolysis.^4^ Many molecules are identified as intracellular second messengers able to mediate cellular responses involved in muscle atrophy, including reactive oxygen species (ROS), cAMP, and calcium.^4^, ^5^, ^6^ Interestingly, in the last decades, iron has also been proposed as a potential second messenger or cofactor involved in skeletal muscle wasting and sarcopenia.^7^, ^8^, ^9^

Iron is a vital element that can accept and donate electrons readily and is involved in many essential cellular processes associated with basic functions like oxygen transport and enzymatic activity.^10^ Cell iron homeostasis is tightly tuned, especially through the iron response element–iron regulatory protein (IRE–IRP) system that modulates the mRNA stability or the synthesis of proteins regulating its availability and traffic, according to the cell iron levels.^11^ In some pathological conditions, such as hereditary haemochromatosis, repeated blood transfusions, or excess oral iron intake, systemic iron homeostasis is dysregulated and intracellular iron overload may occur. These conditions can promote the formation of ROS through the Fenton reaction leading to the oxidation of lipids, proteins, and DNA.^12^ Thus, iron overload impacts the function of several organs, including the liver,^13^ pancreas,^14^ bone,^15^ and heart.^16^ The development of organ iron overload in those conditions may involve the formation of abnormal biochemical forms of iron in plasma, such as the non‐transferrin bound iron (NTBI), which is linked to low molecular compounds in plasma and is avidly taken up by organs, especially by liver, heart, and pancreas.^17^

As ~10% of total body iron is stored in skeletal muscle, the impact of iron overload on muscle phenotype has also been explored during the last decade.^18^ Previous studies reported a strong correlation between muscle iron accumulation occurring with ageing, ROS production, and muscle wasting.^19^, ^20^, ^21^, ^22^ Moreover, iron overload‐induced skeletal muscle atrophy has been associated with ROS‐mediated ubiquitin–proteasome system activation.^22^, ^23^, ^24^ However, the iron supplementation used in these studies causes systemic and cellular iron overload,^22^, ^23^, ^24^ much higher than that observed under pathophysiological conditions, especially in patients with haemochromatosis.^25^

Although extra‐physiological iron overload is known to induce skeletal muscle wasting, the impacts of iron excess close to pathophysiology levels on skeletal muscle mass and cellular mechanisms involved in iron homeostasis remain unclear. Therefore, the main objective of the present study was to determine the impact of pathophysiological iron overload on skeletal muscle structure and cellular adaptations. In this study, we characterized structural and metabolic adaptations occurring in skeletal muscles from iron‐exposed C2C12 myotubes and iron dextran‐supplemented mice. Our data support that, in conditions close to pathological situations in humans, muscle iron accumulation does not induce muscle atrophy, due to cellular adaptations, which differ according to muscle fibre type.

## Material and methods

Animal experiments were approved by the Animal Experimentation Ethics Committee in accordance with the recommendations of the Guide for the Care and Use of Laboratory Animals (National Institutes of Health Publication No. 85‐23, Revised 1996) and according to the European directives (86/609/European Economic Community).

### Animal experiments

Two‐month‐old C57BL/6 male mice obtained from Janvier Labs (Le Genest‐Saint‐Isle, France) were randomly divided in the following two experimental groups: a group treated with one subcutaneous injection of iron dextran (1 g/kg in 200 μL) (IRON, *n* = 10), as previously reported,^26^, ^27^ and a control group that received one subcutaneous injection (200 μL) of dextran vehicle only (VEH, *n* = 10). All animals were maintained at the ARCHE facilities (UMS Biosit, Rennes, France) with standard conditions for temperature (19°C), atmosphere, and light (12/12 h). The animals had free access to tap water and standard food (2018 Teklad 18% Protein Rodent Diet with an iron content of 200 mg/kg). Four months after the injection, mice were deeply anaesthetized with isoflurane (2–3%). Intracardiac blood was collected in dry tubes, and the mice were euthanized by cardiac exsanguination. Blood was then centrifuged (1500 *g* for 10 min) for serum isolation. Soleus, gastrocnemius muscles, and liver were harvested, weighed, and frozen in liquid nitrogen or fixed in 4% paraformaldehyde.

### Serum analyses

Serum iron and unsaturated iron‐binding capacity (UIBC) were measured in the biochemistry laboratory (Rennes Pontchaillou Hospital) on Roche Cobas^®^ 8000 analyser (Cobas Reagents 03183696 122 and 04536355190, respectively, Roche Diagnostics, Meylan, France). Serum transferrin saturation was calculated as [serum iron∕(serum iron + UIBC)] × 100 and expressed as percentage.

### Iron quantifications in liver and skeletal muscles

Hepatic and skeletal muscle iron pool has been quantified on the ‘Analyse Elémentaire et Métabolisme des Métaux’ (AEM2) Platform (Université de Rennes 1—Centre Hospitalier Universitaire). Frozen skeletal muscle and liver samples were desiccated at 120°C for 15 h in an overnight. They were weighed and mineralized according to a previous protocol.^26^ Briefly, nitric acid solution (Optima Grade, Thermo Fisher Scientific, Waltham, MA, USA) was added to dried samples, and then the tubes were placed in a MARS6 microwave (CEM, Matthews, NC, USA) with a temperature maintained at 180°C. Iron (^56^Fe) was quantified by inductively coupled plasma mass spectrometry on an X‐Series IO from Thermo Fisher Scientific equipped with collision cell technology (ÆM2 platform). Calibration ranges were prepared using a multi‐element calibrator solution (Plasma Cal, SCP SCIENCE, Baie‐D'Urfe, QC, Canada).

### Histological analysis

Gastrocnemius muscles were fixed in paraformaldehyde 4% during 24 h and then embedded in paraffin. Serial transverse sections of 4 μm were cut from the wider part of each sample using a Leica microtome and were mounted on glass slides (three sections per slide). Muscle sections were then stained using Gomori's method to detect reticulin fibres.^28^ All stained sections were scanned (×20 objective) to measure the fibre cross‐sectional area from at least 300 fibres per muscle using the Image‐Pro Plus software 6.0 (Media Cybernetics, Inc.).

### Muscle fibre phenotype

The muscle fibre phenotype was determined by assessing the presence of the different myosin heavy chain (MHC) isoforms (slow MHC I and fast MHC IOa, IOd/x, and IOb isoforms), as previously described.^29^, ^30^ Protein samples (6 μg) were separated on high‐glycerol‐containing (30%) gels with an acrylamide‐to‐bis‐acrylamide ratio of 50:1 for the separating gel (8% total acrylamide, pH 8.8) and for the stacking gel (4% total acrylamide, pH 6.8) using a Mini‐PROTEAN IO dual slab cell apparatus (Bio‐Rad Laboratories, Inc., Hercules, CA, USA). After electrophoresis (at 140 V in a refrigerated room at 4°C for 22 h), gels were stained with Coomassie blue (IRDye^®^ Blue Protein Stain, LI‐COR Biosciences, Lincoln, NE, USA), and the different MHC isoforms were identified according to their electrophoretic mobility pattern.

### Cell culture

C2C12 murine myoblasts obtained from ECACC (91031101) were grown in Dulbecco's modified Eagle's medium (25 mM glucose, Gibco, USA) supplemented with 10% foetal bovine serum (Gibco) and 1% penicillin/streptomycin/glutamine (Gibco) in a humidified atmosphere with 5% CO_2_ at 37°C. When cells reached 70–75% confluence, the medium was changed to Dulbecco's modified Eagle's medium supplemented with 2% horse serum (Gibco) to induce differentiation. The induction medium was changed every day during the next 5 days. Then, differentiated myotubes were treated in serum‐free medium with 10 and 50 μM of ferric ammonium citrate (FAC) for different times depending on the experiments.

### Measurement of myotube size

After 72 h of FAC treatment, myotubes were fixed in 4% paraformaldehyde for 10 min, washed in phosphate‐buffered saline (PBS) 1×, and then permeabilized with PBS–bovine serum albumin (BSA) 2%–saponin 0.2% for 30 min. Cells were then incubated at room temperature with an anti‐pan MHC antibody (MyH, sc‐20641, 1:200; Biotechnology) for 2 h and washed in PBS 1× before staining with a secondary goat anti‐rabbit immunoglobulin (Alexa Fluor 488, 1:2000 dilution, Cell Signaling) for 1 h. The images were visualized with a fluorescence microscope (Axio Observer Z1, Zeiss, Germany). Myotube size was quantified by measuring diameter of 200 myotubes for each condition from 10 random fields at ×20 magnification using ImageJ software. Three diameter measurements were taken along the length of each myotube, and the mean diameter was calculated.

### RNA extraction and reverse transcription–real‐time PCR

After 24 h of FAC exposure, myotubes were washed in cold PBS and centrifuged, and the cell pellet was then frozen in liquid nitrogen. Skeletal muscles from *in vivo* experiments were ground in liquid nitrogen, and the obtained powder was used to perform experiments. Total RNA was extracted using TRIzol^®^ reagent according to the manufacturer's protocol (Sigma‐Aldrich). RNA extraction was directly realized on frozen C2C12. RNA amounts were determined by NanoDrop spectrophotometer, and RNA quality was controlled on 1.2% agarose gel using the FlashGel electrophoresis system (Lonza). Reverse transcription reaction (RT) was carried out on 1 μg of total RNA (IScript reverse transcription, 170‐8840). Then, real‐time PCR experiments were performed on RT products in a final volume of 10 μL containing 5 μL of cDNA (diluted at 1/5), 0.2 μL of primers (10 μM), and 4.8 μL of SYBR^®^ Green Supermix (1725271, Bio‐Rad Laboratories, Inc.). Experiments were monitored in CFX Real‐Time machine (Bio‐Rad Laboratories, Inc.). The expression of target genes was normalized to reference genes, and the relative expression was calculated using the ΔΔCt method. Primer sequences are listed in *Table* 1. The reference genes were ribosomal protein L4 (*Rpl4*) and ribosomal protein L19 (*Rpl19*).

**Table 1:** Sequences of primers (5′–3′) used for RT‐qPCR

Gene	Reference	Forward	Reverse
*Aconitase 1*	NM_007386.2	CAGAAGGGCAGACAGTTTACAGA	GAACTCCCAGACCGTCAATCA
*Atg5*	XM_011243108.3	GGCACACCCCTGAAATGG	TCCTTGGATGGACAGTGTAGAAG
*Atg7*	NM_001253717.1	GGCTAGGACACTGATGGGCTG	GAACCCTCTGGCATTCACTCC
*Cyc1*	NM_025567.3	GGACCACACCAGCATTCG	CCTTGGCTTCTTCCTCCGT
*Dmt1*	XM_006520579.3	CGTCAGTATCCCAAGGTCCCAC	CGCTTCCAGCTTCCGCAA
*Fpn*	NM_016917.2	CCAGTGTCCCCAACTACCAA	GTCACCGTCAAATCAAAGGA
*FtH*	NM_010239.2	GCTGCCATCAACCGCCA	GCAAAGTTCTTCAGAGCCACATC
*Myoglobin*	NM_001164048.1	GGAAGTCCTCATCGGTCTGTTTA	CCATGCTTCTTCAGGTCCTCTG
*Rpl4*	NM_024212.4	CTTCTGGGCCTGCTGTTCA	CCAGCCTACTCATTGGGATCA
*Rpl19*	NM_001159483.1	GAAGGTCAAAGGGAATGTGTTCA	CCTTGTCTGCCTTCAGCTTGT
*Tfr1*	NM_011638.4	TGATTGTTAGAGCAGGGGAAA	ATGACTGAGATGGCGGAAAC
*Zip14*	XM_006252270.2	GTGCTCACTTACTTCATCGCCT	TATGTCCGTGATGGTGCTCG

### Cytosolic protein extraction

After 72 h of FAC exposure, myotubes were washed in cold PBS and centrifuged, and the cell pellet was then frozen in liquid nitrogen. Skeletal muscles from *in vivo* experiments were ground in liquid nitrogen, and the obtained powder was used to perform experiments. Cytosolic protein extraction was performed from soleus, gastrocnemius, and C2C12 in cold lysis buffer containing 10.0 mM Tris·HCl, pH 7.4, 0.5 M sucrose, 50.0 mM NaCl, 5.0 mM EDTA, 30.0 mM Na_4_P_2_O_7_, 1% NP‐40, 0.25% sodium deoxycholate, 50.0 mM NaF, 100.0 M sodium orthovanadate, and protease inhibitor cocktail (5 L/mL, P8340; Sigma, St. Louis, MO, USA). The samples were homogenized using a Polytron homogenizer at 4°C. Each sample was then incubated on ice for 30 min followed by 10 s of sonication. The homogenates were then transferred to microcentrifuge tubes and centrifuged at 12 000 *g* for 12 min at 4°C. The protein concentration of the supernatant was determined by a Lowry assay using bovine serum albumin as a standard.

### Western blotting

Samples were diluted in SDS‐PAGE sample buffer (50 mM Tris·HCl, pH 6.8, 2% SDS, 10% glycerol, 5% mercaptoethanol, and 0.1% bromophenol blue) and heated 5 min at 95°C until analyses. Samples containing 50 μg of proteins were resolved on 10% or 12.5% SDS‐PAGE. The proteins were transferred at 240 mA for 90 min onto a 0.2 μm nitrocellulose membrane. Membranes were blocked with 5% BSA or non‐fat dry milk in Tris‐buffered saline/0.05% Tween 20 (TBST) for 1 h at room temperature. Membranes were incubated overnight at 4°C with appropriate primary antibodies (*Table* 2). Thereafter, membranes were washed with TBST and incubated for 1 h at room temperature with infrared dye‐conjugated secondary antibodies (LI‐COR Biosciences). After being washed, blots were captured using the Odyssey Imaging System (LI‐COR Biosciences). All blots were scanned, and densitometry analysis of the bands was conducted using GS‐800 imaging densitometer and Quantity One software (Bio‐Rad Laboratories, Inc.). All blots were corrected for loading based on the HSC70 expression.

**Table 2:** List of primary antibodies used for western blotting

Protein	Reference	Source	Dilution
4‐HNE	Abcam, ab46545	Rabbit	1/1000
COXIV	Cell Signaling, 4850	Rabbit	1/1000
Cytochrome *c*	Santa Cruz, sc13156	Mouse	1/2000
DMT1	Santa Cruz, sc166884	Mouse	1/200
FtH	Abcam, ab65080	Rabbit	1/1000
HSC70	Santa Cruz, sc7298	Mouse	1/5000
MHC	Santa Cruz, sc‐25619	Rabbit	1/200
MurF1	Abcam, ab183094	Rabbit	1/1000
p‐4EBP1^Thr37/46^	Cell Signaling, 2855	Rabbit	1/1000
4EBP1	Cell Signaling, 9644	Rabbit	1/1000
p‐Akt^Ser473^	Cell Signaling, 9271	Rabbit	1/1000
Akt	Cell Signaling, 9272	Rabbit	1/1000
NFκB p65	Cell Signaling, 4764	Rabbit	1/1000
TfR1	Cell Signaling, 13113	Rabbit	1/1000
Ubiquitin (Ub)	Cell Signaling, 3933	Rabbit	1/1000

### Protein carbonylation

Quantification of protein carbonyl levels was performed using an OxyBlot^®^ kit according to the manufacturer's protocol (S7150, Millipore, Île‐de‐France, France). Cytosolic protein extracts from soleus and gastrocnemius (15 μg) were derivatized with 1% DNPH for 15 min at RT and then separated on 10% SDS‐PAGE. After transfer, membranes were blocked successively incubated in 1% BSA in PBS/0.05% Tween 20 (PBST) during 1 h and then overnight at 4°C with anti‐DNP primary antibody (1:200) supplied in the OxyBlot kit. Thereafter, membranes were washed with PBST and incubated for 1 h at room temperature with infrared dye‐conjugated secondary antibodies (LI‐COR Biosciences). After being washed, blots were captured using the Odyssey Imaging System (LI‐COR Biosciences).

### 4‐Hydroxy‐2‐nonenal analysis

Detection of 4‐hydroxy‐2‐nonenal (4‐HNE) protein adducts was performed as previously described.^31^ Briefly, membranes were incubated in a solution of 250 mM sodium borohydride in 100 mM MOPS, for 30 min at RT, immediately after transfer. Membranes were blocked with 1% BSA PBST during 1 h and then incubated overnight at 4°C with the primary antibody (*Table* 2). Thereafter, membranes were washed with PBST and incubated for 1 h at room temperature with infrared dye‐conjugated secondary antibodies (LI‐COR Biosciences). After being washed, blots were captured using the Odyssey Imaging System (LI‐COR Biosciences).

### Statistical analyses

All data are presented as the mean ± standard deviation. The normality of each distribution and homogeneity of variance were assessed with the Shapiro–Wilk and Levene tests, respectively. Student's *t*‐test was then performed to explore the effects of iron supplementation. The Mann–Whitney *U* test was chosen when the normality and/or equal variance test failed. A two‐way ANOVA was performed to assess the effects of both iron supplementation and typology on muscle iron content. Significant interaction effects were analysed with Tukey's *post hoc* test. For C2C12 experiments, Kruskal–Wallis and Dunn's *post hoc* tests have been performed. For all statistical analyses, the significance level was set at 0.05. Data were analysed using the statistical package GraphPad Prism Version 7.00 for Windows (GraphPad Software, La Jolla, CA, USA).

## Results

### Responses of differentiated myotubes to ferric ammonium citrate treatment

**Figure 1:**
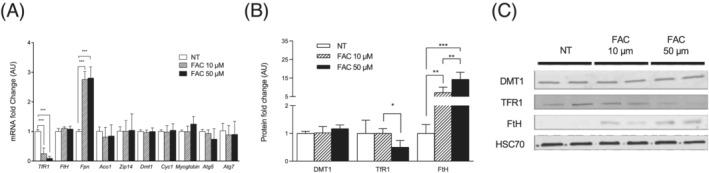
Responses of C2C12 differentiated myotubes to FAC treatment. Differentiated C2C12 myotubes were exposed to various concentrations of FAC (10 and 50 μM) in serum‐free medium for 24 h (qPCR analyses) or 72 h (western blot analyses). (A) Iron metabolism gene expression in response to FAC 10 and 50 μM. (B) Iron metabolism protein expression in response to FAC 10 and 50 μM. (C) Representative blotting images used for the quantification shown in (B). Values are the mean ± SD. The experiments were performed on three independent replicates of three dependent samples for each condition. Significance was checked using Kruskal–Wallis and Dunn's multiple comparisons. Significant differences between conditions (*P < 0.05, **P < 0.01, and ***P < 0.001, respectively). *Aco1*, aconitase 1; *Atg5*, autophagy‐related 5; *Atg7*, autophagy‐related 7; *Cyc1*, cytochrome *c*; *Dmt1*, divalent metal transporter 1; *Fpn*, ferroportin; *FtH*, ferritin heavy chain; *HSC70*, heat shock cognate 71 kDa protein; *TfR1*, transferrin receptor 1; *Zip14*, ZRT/IRT‐like protein 14.

*Figure* 1 presents the expression of iron metabolism genes and proteins in differentiated myotubes when exposed to 10 or 50 μM FAC, a form of NTBI. A 72 h treatment with FAC induces an increase in ferritin protein expression in myotubes. This effect seems to be dose dependent (7‐fold and 13‐fold increase for 10 and 50 μM, *P* = 0.002 and *P* < 0.001, respectively, *Figure* 1B and 1C). A concomitant gradual decrease in mRNA TfR1 levels is also detected (−76% and −91% for 10 and 50 μM, *P* < 0.001, *Figure* 1A). Only 50 μM FAC induced a significant reduction in TfR1 protein levels (−50%, *P* = 0.031, *Figure* 1B and 1C). Interestingly, *Fpn* mRNA level significantly increases for 10 and 50 μM FAC (2.8‐fold change for both, *P* < 0.001, *Figure* 1A). Finally, mRNA coding for other iron transport proteins (i.e. *DMT1* and *Zip14*), iron storage (i.e. *FtH*), iron–sulfur proteins (i.e. *Aco1* and *Cyt c*), myoglobin, and autophagy‐related proteins (i.e. *Atg5* and *Atg7*) remains unaffected by exposure to FAC.

### Impact of ferric ammonium citrate treatment on myotube size and signalling pathway involved in muscle mass regulation and mitochondrial function

**Figure 2:**
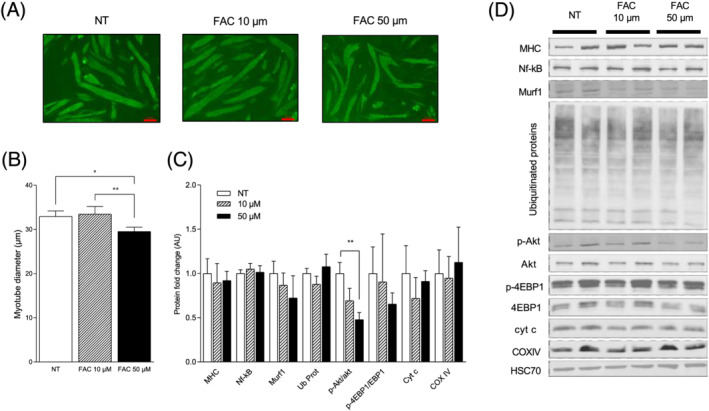
Impact of FAC treatment on myotube size and signalling pathway involved in muscle mass regulation and mitochondrial function. Differentiated C2C12 myotubes were exposed to various concentrations of FAC (10 and 50 μM) in serum‐free medium for 72 h. (A) Representative morphology of myotubes at 72 h after vehicle or FAC treatment. (B) Quantitative analysis of myotube diameter. (C) Protein expression or activation of key proteins involved in proteolysis, protein synthesis, and mitochondrial function in response to FAC 10 and 50 μM. (D) Representative blotting images used for the quantification shown in (B). Values are the mean ± SD. The experiments were performed on three independent replicates of three dependent samples for each condition. Significance was checked using Kruskal–Wallis and Dunn's multiple comparisons. Significant differences between conditions (^**^ *P* < 0.01). *4EBP1*, eukaryotic translation initiation factor 4E‐binding protein 1; *Akt*, protein kinase B; *COXIV*, cytochrome *c* oxidase IV; *Cyt c*, cytochrome *c*; *HSC70*, heat shock cognate 71 kDa protein; *Murf1*, muscle RING‐finger protein‐1; *MHC*, myosin heavy chain; *Nf‐κB*, nuclear factor‐κB; *Ub Prot*, polyubiquitinated proteins.

In differentiated myotubes, we observed that 10 μM FAC does not affect myotube size, but that 50 μM FAC significantly decreases this parameter compared with non‐treated myotubes (−11%, *P* = 0.018, *Figure* 2A and 2B). MHC protein content remains unchanged under 10 and 50 μM (*Figure* 2C and 2D). FAC exposures are not associated to significant changes in protein expression involved in proteolysis signalling pathways (i.e. NF‐κB, MurF1, and ubiquitinated proteins) or in mitochondrial function (i.e. cyt c and COXIV). Finally, we found that the Akt activation (i.e. pAkt/Akt) is significantly reduced in myotubes exposed to 50 μM FAC (−53%, *P* = 0.0035, *Figure* 2C and 2D).

### Iron metabolism differs between oxidative and glycolytic skeletal muscles

**Figure 3:**
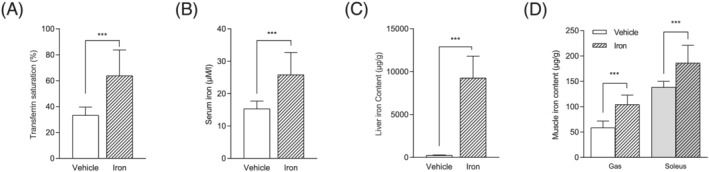
Iron supplementation causes systemic and muscle iron overload. (A) Transferrin saturation. (B) Serum iron concentrations. (C) Liver iron concentrations. (D) Soleus and gastrocnemius iron concentration. Values are the mean ± SD (*n* = 12 per group). Significance was checked using Mann–Whitney *U* test, Student's unpaired *t*‐test, or two‐way ANOVA. Significant differences between conditions (^***^ *P* < 0.001).

**Figure 4:**
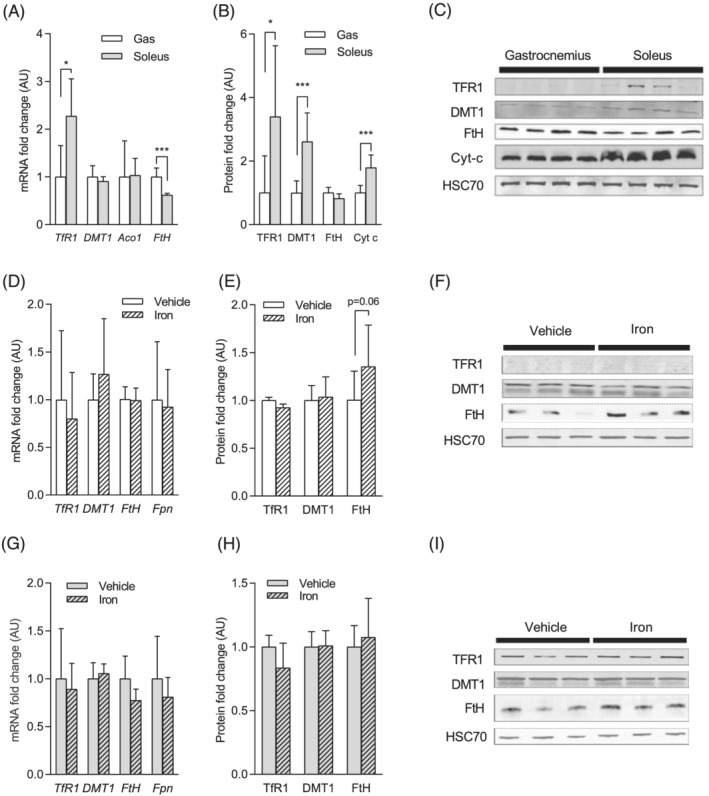
Muscle adaptations of iron metabolism under iron supplementation. (A) Iron metabolism protein gene expression in gastrocnemius and soleus. (B) Iron metabolism protein expression in gastrocnemius and soleus. (C) Representative blotting images used for the quantification shown in (B). (D) Iron metabolism protein gene expression in gastrocnemius in response to iron dextran treatment. (E) Iron metabolism protein expression in gastrocnemius in response to iron dextran treatment. (F) Representative blotting images used for the quantification shown in (E). (G) Iron metabolism protein gene expression in soleus in response to iron dextran treatment. (H) Iron metabolism protein expression in soleus in response to iron dextran treatment. (I) Representative blotting images used for the quantification shown in (H). Values are the mean ± SD (*n* = 6–12 per group). Significance was checked using Mann–Whitney *U* test or Student's unpaired *t*‐test. *DMT1*, divalent metal transporter 1; *Fpn*, ferroportin; *FtH*, ferritin heavy chain; *HSC70*, heat shock cognate 71 kDa protein; *TfR1*, *transferrin receptor 1*.

We first observed that iron content is significantly higher in the soleus compared with the gastrocnemius in both treated and non‐treated mice (+130% and +78%, *P* < 0.001, respectively, *Figure* 3D). We also found that mRNA and protein levels of TfR1 are significantly higher in the soleus compared with the gastrocnemius (+127% and +239%, respectively, *P* < 0.05, *Figure* 4A–4C). *Dmt1* mRNA levels do not differ between the two muscles, whereas DMT1 protein levels are significantly higher in the soleus compared with the gastrocnemius (+161%, *P* < 0.001, *Figure* 4B and 4C). On contrary, *FtH* mRNA levels are significantly lower in the soleus compared with the gastrocnemius (−38%, *P* < 0.001, *Figure* 4A), whereas FtH protein levels do not differ between these skeletal muscles (*Figure* 4B and 4C). Whereas *Aco1* mRNA levels do not differ between soleus and gastrocnemius, the protein levels of cytochrome *c* are significantly higher in the soleus compared with the gastrocnemius (+59%, *P* < 0.001, *Figure* 4B and 4C).

### Iron supplementation causes iron overload in oxidative and glycolytic skeletal muscle

Four months after iron dextran injection, transferrin saturation and serum iron concentration significantly increase compared with the vehicle group (+68%, *P* < 0.001 and +92%, *P* < 0.001, respectively, *Figure* 3A and 3B). The results are associated with a very high increase of iron content in the liver of iron‐treated mice compared with non‐treated mice (+3944%; *P* < 0.001, *Figure* 3C). Iron supplementation also induces significant increases of iron content in both the soleus (i.e. oxidative) and gastrocnemius (i.e. glycolytic) muscles (+34%; *P* = 0.002 and +79%, *P* < 0.001, respectively, *Figure* 3D).

### Muscle adaptations of iron metabolism under iron supplementation

**Figure 5:**
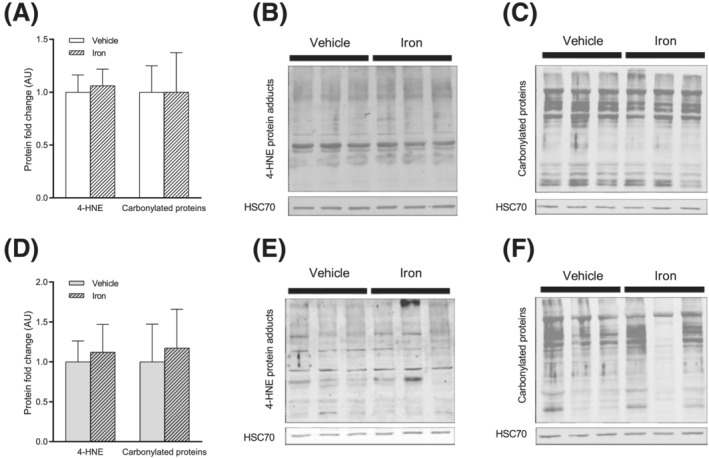
Iron supplementation does not induce oxidative damage in skeletal muscle. (A) 4‐HNE protein adducts and carbonylated proteins in gastrocnemius. (B) Representative blotting images for 4‐HNE protein adducts in gastrocnemius. (C) Representative blotting images for carbonylated proteins in gastrocnemius. (D) 4‐HNE protein adducts and carbonylated proteins in soleus. (E) Representative blotting images for 4‐HNE protein adducts in soleus. (F) Representative blotting images for carbonylated proteins in soleus. Values are the mean ± SD (*n* = 7–9 per group). Significance was checked using Mann–Whitney *U* test or Student's unpaired *t*‐test.

We observed that iron dextran injection tends to increase the iron storage ferritin protein expression in the gastrocnemius after 4 months (+36%; *P* = 0.06, *Figure* 4E and 4F), whereas it remains unchanged in the soleus (*Figure* 4H and 4I). mRNA and protein levels of other iron metabolism proteins (i.e. TfR1, DMT1, and FPN) remain unaffected both in the soleus and in the gastrocnemius in iron‐treated animals (*Figure* 4D–4I). 4‐HNE protein adducts and carbonylated proteins levels, quantified in order to assess oxidative damage in soleus and gastrocnemius, are not significantly affected in gastrocnemius (*Figure* 5A–5C) and soleus (*Figure* 5D–5F).

### Iron supplementation does not impact skeletal muscle structure in mice

**Figure 6:**
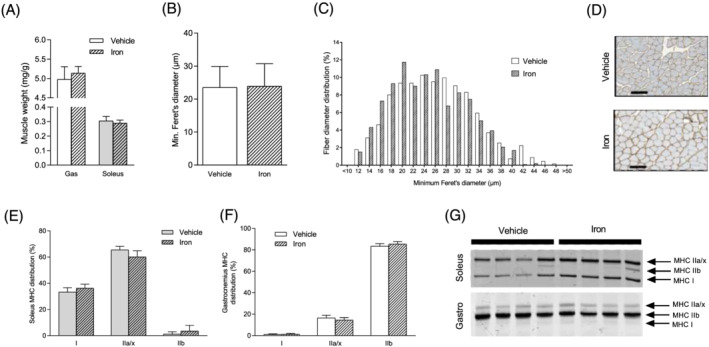
Iron supplementation does not impact skeletal muscle structure in mice. (A) Gastrocnemius and soleus weight‐to‐body weight ratio. (B) Minimal Feret's diameter of muscle fibres from gastrocnemius muscle. (C) Frequency distribution of muscle fibre's diameter in the gastrocnemius muscle. (D) Laminin representative staining of skeletal muscle section. Black scale bar corresponds to 100 μm. (E) Distribution of MHC isoforms in soleus muscle. (F) Distribution of MHC isoforms in gastrocnemius muscles. (G) Representative electrophoresis of MHC isoforms in soleus and gastrocnemius muscles. Values are the mean ± SD (*n* = 6–12 per group). Significance was checked using Mann–Whitney *U* test or Student's unpaired *t*‐test.

Four months after iron dextran injection, the soleus and gastrocnemius weight‐to‐body ratio remain unchanged in iron‐treated mice compared with non‐treated mice (*Figure* 6A). Similarly, iron supplementation does not affect gastrocnemius muscle fibre diameter (*Figure* 6B–6D). MHC distribution in the soleus (*Figure* 6E and 6G) and gastrocnemius muscles (*Figure* 6F and 6G) does not significantly change in iron‐treated mice.

## Discussion

The aim of the present study was to investigate whether iron overload reflecting pathophysiological conditions impacts skeletal muscle structure, cellular mechanisms regulating iron metabolism, or muscle atrophy. In response to iron overload close to clinically relevant levels, our data contrast with previous studies,^23^, ^24^, ^32^, ^33^ as excess iron did not induce muscle atrophy. Indeed, FAC‐treated myotubes and iron dextran supplementation in mice induced iron accumulation in cells and skeletal muscle, but neither muscle atrophy nor activation of key signalling pathways involved in proteolysis. These data suggest that skeletal muscles can protect themselves from excess iron and its related deleterious effects through cellular mechanisms, which depend on muscle fibre type.

In basal condition, the 2.5‐fold higher level of iron content we reported in the mice soleus compared with the gastrocnemius is in accordance with the metabolic phenotype of these two skeletal muscles. The soleus is an oxidative skeletal muscle dense in mitochondria with slow contractile properties that requires more energy, and therefore iron, compared with a fast‐glycolytic muscle such as the gastrocnemius.^34^, ^35^ This higher demand for iron of this muscle is reflected by the higher TfR1 protein levels in the soleus muscle, suggesting a potential link between oxidative metabolism and iron import. Altogether, the data suggest that mitochondria could drive the uptake of transferrin iron to meet the higher cellular needs of this oxidative muscle.^36^ Despite the higher level of ferritin mRNA levels in the gastrocnemius compared with the soleus, the ferritin protein itself is expressed at a similar level in both muscles. These findings reflect the fact that the cellular machinery may perfectly balance the iron uptake according to cellular iron needs. The IRP–IRE system is one of the main intracellular iron metabolism regulators due to its role in the control of transferrin receptor 1 mRNA stability and ferritin protein translation in a coordinated manner through interactions with IRE sequences located in 3′ and 5′ untranslated mRNA region, respectively.^11^ The increase of TfR1 protein levels reported in the soleus could then likely involve the IRE–IRP system. Notably in the gastrocnemius, there was no increase in ferritin protein levels despite the ferritin mRNA levels being higher. Again, such findings could indicate that the IRE–IRP system neutralizes the impact of the higher ferritin mRNA level, the synthesis of this iron storage protein being in equilibrium with the lower cellular iron content in this glycolytic muscle. In myotubes exposed to iron, we observed a decrease of TfR1 mRNA levels combined with an increase of ferritin protein levels that, together with dose dependency, strongly support a role of the IRE–IRP system in the control of iron metabolism in muscle cells.

Despite excess iron, we did not observe muscle atrophy in either *in vitro* or *in vivo* conditions. Indeed, differentiated myotubes are protected from atrophy under 10 μM FAC, corresponding to levels of free iron close to serum NTBI levels observed in hereditary haemochromatosis or β‐thalassemic patients (i.e. between 1 and 10 μM).^37^, ^38^ We also exposed myotubes higher doses of FAC (i.e. 50 μM), which is in a range far above the NTBI values observed in iron overloaded patients, even in thalassemia.^38^, ^39^ In response to this very high dose, we found a significant slight reduction of myotube size. Despite the fact that the content of proteins involved in proteolysis pathways (i.e. NF‐κB, MurF1, and ubiquitinated proteins) remained unchanged, a significant decrease of Akt activation level was found when exposing myotubes to 50 μM FAC. A similar observation was previously reported by others in differentiated myotubes exposed to higher doses of iron (i.e. 100 μM iron sulfate).^23^ Akt is a protein kinase playing a central role in muscle metabolism by regulating insulin‐dependent glucose uptake and protein synthesis.^40^, ^41^ In contrast to a previous study,^23^ our data suggest that the atrophic effect of iron excess is only found (i) with very high, and not clinically relevant levels of NTBI, and (ii) seems more related to an inhibition of anabolic processes more than an activation of catabolic pathway. Such a hypothesis should be explored in further experiments. Together, our data suggest that pathophysiological iron overload does not induce skeletal muscle atrophy *in vitro* probably due the IRE–IRP system protecting against iron excess.

The parenteral iron supplementation that we performed in mice has been previously shown to lead to an iron overload in both parenchymal cells and macrophages.^26^, ^27^ In accordance to these data, we report blood iron parameters in iron‐treated mice close to those observed in hereditary haemochromatosis patients (i.e. transferrin saturation >55%, serum iron levels >27 μmol/L, and liver iron content >8000–10 000 μg/g).^42^, ^43^ These results are also in accordance with the range of serum iron levels and transferrin saturation we also observed in 6‐month‐old *Hfe* ^*−/−*^ mice (Supporting Information, *Figure* S1). In parallel, the muscle iron concentrations increase both in the soleus and in the gastrocnemius of iron supplemented animals, whatever the contractile and metabolic phenotype. Notably, these elevations of iron concentrations are associated to an increase of ferritin protein levels in gastrocnemius, but not in soleus. Interestingly, we report similar increase of ferritin protein levels in gastrocnemius of *Hfe* ^−/−^ mice, whereas these levels remain also unaffected in soleus of those animals (*Figure* S1). Altogether, such data support that mechanisms of adaptation to chronic iron overload may differ depending on muscle fibre type.

The ability of labile cytosolic ‘free’ iron to generate oxidative damage is currently considered as the main mechanism proposed to explain whether iron excess promotes muscle atrophy.^21^, ^23^, ^24^ However, despite elevations of muscle iron concentrations in iron supplemented animals, we observed that neither 4‐HNE protein adducts nor carbonylated proteins levels increase in soleus and gastrocnemius. These data suggest that skeletal muscles, whatever its typology and metabolism, can adapt to an iron excess close to situations observed in humans and protect themselves from potential oxidative damage. In accordance with these findings and our *in vitro* results, no reduction in muscle weight and fibre size compared with control animals was observed in iron‐treated mice. Such data contrast with previous studies reporting that iron overload promotes skeletal muscle atrophy in mice.^23^, ^24^, ^32^ However, experimental protocols used in those studies induced a systemic and muscle iron overload largely beyond the classical findings observed in pathophysiological conditions. Here, we used an experimental murine model iron overload,^26^, ^27^ which involved injecting a unique dose of iron dextran to animals (1 g/kg), corresponding to an absolute dose of iron range from 25 to 35 mg. Four months after the injection, we reported a clinical picture of a high iron overload with blood parameters similar to those observed in humans.^42^, ^43^ On contrary, Reardon *et al*. injected iron dextran several times over a month to a total of dose of 200 mg,^24^ whereas Ikeda *et al*. injected a total of 140 mg in only 2 weeks of treatment.^23^ As the body's iron metabolism functions in a very closed system with very low daily losses, these treatments promote extra‐physiological iron overloads that are relevant for studying intoxication or poisoning, but not for extrapolations in chronic iron overload condition. Thus, Reardon *et al*. observed plasma iron levels around 290 μmol/L in iron‐treated mice,^24^ which was 10‐fold higher than the levels reported in our mice and is never found in humans. In addition, our findings are consistent with the recent study of Liang *et al*. reporting a four‐fold increase of iron content in the soleus without reduction of muscle weight after a 2 week treatment with a total of 40 mg of iron dextran injected in animals, which is closer to our doses.^44^ Interestingly, a significant reduction of soleus maximal muscle strength was reported in this study independently from a reduction of muscle mass.^44^ A disturbance in the muscle redox balance and sarcoplasmic reticulum Ca^2+^ release was proposed as potential mechanisms to explain this reduction of muscle strength.^44^ In our study, we also questioned the impact of iron excess on muscle contractile phenotype recognized to affect maximal muscle strength independently from muscle mass.^45^ We found that MHC distribution remains unchanged after 4 months in both the soleus and gastrocnemius muscles, suggesting that contractile phenotype would not be impacted by iron excess.

In summary, our data highlight that despite the distinct iron metabolism phenotype of oxidative and glycolytic skeletal muscles in a basal state, pathophysiological iron overload does not cause skeletal muscle atrophy in mice. Therefore, in a clinically relevant condition, skeletal muscles seem able to protect themselves from the excess iron and its related deleterious effects. This study opens new perspectives to improve the management of patients exhibiting skeletal muscle wasting conditions in which muscle iron accumulation may occur, including ageing, myopathy, and disuse.

## Funding

This study was supported by grants from the French Society for Rheumatology [Société Française de Rhumatologie (SFR)] and the French Centre National d'Etudes Spatiales (CNES).

## Conflict of interest

No conflict of interest, financial or otherwise, is declared by the authors.

## Supporting information


**Figure S1.** Iron metabolism parameters in 6 months old wild‐type (WT) and Hfe^−/−^ mice A) Transferrin saturation. B) Serum iron levels. C) Liver iron concentrations. D) Ferritin H protein levels. E) Representative Western Blot of ferritin protein expression. Values are the mean ± SD (*n* = 6–12/group). Significance was checked using Mann–Whitney or Student's unpaired t test.
