# Supplementary material for: Oxidative and glycolytic skeletal muscles deploy protective mechanisms to avoid atrophy under pathophysiological iron overload
Source: J Cachexia Sarcopenia Muscle. 2022 Feb 3;13(2):1250–61. doi: 10.1002/jcsm.12897 (PMC8978014; doi:10.1002/jcsm.12897)
Supplement: Supplementary file 1 — Figure S1. Iron metabolism parameters in 6 months old wild‐type (WT) and Hfe−/− mice A) Transferrin saturation. B) Serum iron levels. C) Liver iron concentrations. D) Ferritin H protein levels. E) Representative Western Blot of ferritin protein expression. Values are the mean ± SD (n = 6–12/group). Significance was checked using Mann–Whitney or Student's unpaired t test. [file JCSM-13-1250-s001.docx]

**SUPPLEMENTAL INFORMATION**

**MATERIAL AND METHODS**

**Hereditary hemochromatosis mice (*Hfe*^-/-^).** *Hfe*^−/−^ (n = 12) and *Hfe*^+/+^ (n = 12) C57BL/6 male 6 months old mice were included in this experiment. All animals were maintained at the ARCHE facilities (UMS Biosit, Rennes) with standard conditions for temperature (19°C), atmosphere and light (12h/12h). The animals had free access to tap water and standard food (2018 TEKLAD 18% Protein Rodent Diet with an iron content of 200 mg/kg). At 6 months, mice were deeply anesthetized with isoflurane (2-3%). Intracardiac blood was collected in dry tubes, and the mice were euthanized by cardiac exsanguination. Blood was then centrifuged (1500*g* for 10 min) for serum isolation. Soleus, gastrocnemius muscles and liver were harvested, weighed and frozen in liquid nitrogen.

**SUPPLEMENTAL RESULTS**

**Figure S1.** Iron metabolism parameters in 6 months old wild-type (WT) and Hfe^-/-^ mice A) Transferrin saturation. B) Serum iron levels. C) Liver iron concentrations. D) Ferritin H protein levels. E) Representative Western Blot of ferritin protein expression. Values are the mean ± SD (n=6-12 / group). Signiﬁcance was checked using Mann-Whitney or Student’s unpaired t test.
